# Quantitative determination of al (III) traces in soft drink, pharmaceutical products and biological fluids of kidney failure and alzheimer disease patients using carbon sensor

**DOI:** 10.1038/s41598-025-32308-z

**Published:** 2026-01-06

**Authors:** M. H. Abdel Basset, M. A. Zayed, Eman Yossri Frag

**Affiliations:** Chemistry Department, Faculty of Science, Cairo University, Gamaa Str, 12613 Giza, Egypt

**Keywords:** Carbon paste sensor, Al (III) ion, *p*-chlorophenyl maleanilic acid, Pharmaceutical products, Soft drink and serum samples, Biochemistry, Chemistry

## Abstract

Carbon paste sensor based on *p*-chlorophenyl maleanilic acid (*p*Cl-MA) as an ionophore was synthesized and investigated as an Al(III) ion selective sensor. The developed sensor shows Nernstian slope of 20.32 ± 1.18 mV decade^− 1^ over the working concentration range of 1.0 × 10^− 6^ − 1.0 × 10^− 1^ mol L^− 1^ of Al (III) ion with a detection limit of 3.3 × 10^− 7^ mol L^− 1^. The sensor exhibited a stable potential response in the pH range from 4.0 to 6.0 and a fast response time of 8 s. The prepared sensor indicated a very good selectivity to Al (III) over a variety of cations. The sensor could be easily employed for determination of Al (III) ions in pharmaceutical products, soft drink and biological fluids of kidney failure and Alzheimer disease patients. The reaction mechanism between *p*CL-MA and Al (III) was confirmed by FT-IR spectroscopic analysis, SEM and EDX.

**Supplementary Information:**

The online version contains supplementary material available at 10.1038/s41598-025-32308-z.

## Introduction

 Aluminum is considered as vital and one of the most essential trace elements and its concentration determines whether it will be effective or toxic. To help the vital body physiological functions working well the standard concentration should be underneath an allowable level^1^. Aluminum is the most abundant metal in the earth crust and one of the rich elements in soil minerals and rocks. It is widely used in transport, architecture, household and industrial machines, also in potable water treatment units as a flocculating agent^2–4^. Because of its activity it is rarely found in the metallic form. Aluminum is naturally found in plants, such as potatoes, rice, and mushrooms^5^, the kind of plant, the soil’s acidity, and the irrigation water used, affect impact how much aluminum is found in vegetables and fruits^6^. Also, it is used as food additives like sodium aluminum sulphate, sodium aluminum phosphate, and sodium aluminosilicate which serve as stabilizers and extend the shelf life of most processed foods.

Recent studies reported that the increased concentration of aluminum in bone and in the central nervous system is a main source of neurotoxicity. Also, Aluminum plays a major role in Parkinson’s disease pathology, dialysis and Alzheimer’s disease^7–9^. Humans may ingest a large amount of aluminum because it may be found in food, or transferred to food from kitchen materials, food packaging, foils, cooking utensils, and other product structures, and from food to the human body. Aluminum containers are coated with a protective oxide layer, which is susceptible to erosion due to any physical effect or chemical reaction. Aluminum can seep into the cooked food because of this layer’s deterioration and the use of these components in cooking foods^10^.

Other sources of aluminum are vaccination, antiperspirants, and some drugs, such as toothpaste, antacids, and buffered aspirin^11^ that makes aluminum determination a vital demand. The methods that have been established for aluminum determinations; containing the use of atomic absorption^12–14^, flow injection analysis^15^, inductively coupled plasma atomic emission spectrometry^16^. Most of the previously mentioned methods are costly and require qualified personnel for pre-processing, execution of the analysis, data collection and data analysis and interpretation. Therefore, developing low-cost, easily manipulated methods with a high level of sensitivity is essential. So, ion–selective sensors are considered as alternatives to other analytical methods in the detection of many ionic species. The promising use of sensors in the fields of agriculture, environmental, pharmaceutical and biological analysis is what stimulated that work to develop carbon sensors precise, fast, reproducible and selective for potentiometric investigation of aluminum ions in real samples such as pharmaceutical, canned beverages and biological fluids of kidney failure and Alzheimer disease patients. Sensitivity and selectivity of the ion–selective sensors toward the ion under investigation are controlled by the modifier or electroactive material which considered the main component of the paste. Several chemical compounds were utilized as a modifier (electroactive material) in the preparation of the electrochemical sensors to detect aluminum ions like Schiff bases^17^, crown^18^, polymer^19–21^ however these sensors have many drawback such as time consumed in preparation, long response time, narrow working range, presence of internal solution, the PVC membrane cannot withstand the mechanical stress. The proposed carbon sensor was modified with p-chlorophenyl maleanilic acid (*p*Cl-MA) which is one of the amide group ligands offers two coordination sites O of carboxylic group and N of the amide^22^.

## Experimental

### Reagents and apparatus

The constituents of the paste mixture such as graphite powder and tricresyl phosphate (TCP) were supplied by Aldrich while other plasticizers as dibutyl phthalate (DBP) and o-Nitrophenyloctylether (o-NPOE) were purchased from Fluka and BDH Industries Ltd, respectively. NiCl_2_.6H_2_O, MnCl_2_.4H_2_O, and Al_2_(SO_4_)_3_.16H_2_O were supplied from BDH chemical Ltd. SrCO_3_, Pb(CH_3_COO)_2_.3H_2_O, CaCL_2_ were purchased from laboratory chemicals, Fluka, Fischer scientific, El Madina For chemicals, Adwic, and Scientific Bismarco Office (S.B.O) respectively; while CuCl_2_.2H_2_O, CoCl_2_.6H_2_O, and FeCl_3_.6H_2_O was purchased from LOBA Chemie (laboratory reagents for chemicals). (NH_4_)_6_ Mo_7_ O_24_. 4H_2_O was purchased from Aldrich.

Fresh stock solutions (1.0 × 10^− 1^ mol L^− 1^) of the metal ions were made by dissolving the precisely weighed quantity of metal salts in the proper solvent then complete to the mark with the distilled water to achieve the desired concentration. By serially diluting stock solutions, other diluted solutions (1.0 × 10^− 2^ − 1.0 × 10^− 8^ mol L^− 1^) were created. Throughout the whole work, all solutions were maintained in dark quick-fit bottles in the refrigerator.

The potential measurements were performed using a digital multimeter DT.9205 A. Ag/AgCl reference electrode (Hanna) in conjugation with the developed carbon sensors as working electrode was used. The pH measurements were made using Hanna mV/pH/Temperature meter (Model: pHS – 3CW). Automatic Micropipettes, within volume range of 100–1000 µL (Model: Accupipette USA) was used to accurately measure the small volumes. The FT-IR spectra were carried out on a Perkin-Elmer 1650 spectrometer (4000–400 cm^− 1^) in potassium bromide pellets at the Micro-analytical Center, Cairo University Egypt. The Al (III) content was determined using inductively coupled plasma (ICP) (National research center, Egypt).

### Preparations of real samples

#### Pharmaceutical

Maalox 460 mg Al (OH)_3_ Anti Acid, produced by Sanofi. To 250 mL measuring flask, 1.824 mL of Maalox was added and diluted to the mark using double distilled water to prepare 1.0 × 10^− 2^ mol L^− 1^ solution, then subjected to estimate Al (III) ions content by the carbon sensor and ICP methods.

#### Canned beverages

To estimate the aluminum content in the soft drink which packed in cane and plastic bottle, commercially available canned and bottles products were purchased from a local market in Giza, Egypt, were examined using the carbon sensor and ICP.

**Biological fluids**: Certified serum samples were supplied from El Rahma Hospital (El Rahma Laboratories, Giza, Egypt). Human serum; patient 1: female, Age: 56, patient suffer from chronic kidney failure, Patient 2: male, Age: 63, patient suffer from acute kidney failure and patient 3: male, Age: 65, patient suffer from Alzheimer.

500 µL of the serum sample was mixed with 1mL of 0.2 mol L^−1^ HNO_3_ in 10 mL measuring flask then completed to the mark using double distilled water, then analyzed by the proposed method and ICP^23,24^. To reduce matrix effects, samples were prepared by using HNO_3_ which acting as an oxidizing agent to break down the organic matrix and as a matrix modifier to reduce interferences and diluted with water to obtain a clear sample suitable for analysis^25–28^.

All experiments were performed in compliance with the relevant laws and institutional guidelines (Profession and Ethics Regulations, Resolution of the Ministry of Health and Population No. 238/2003). The protocol was approved by Faculty of Science (Cairo University) institutional committee(s) and all participants provided written informed consent to approve the experiments done with human subjects.

### Preparation of working carbon sensor

CPEs were prepared by grinding 500 mg pure graphite powder and 0.0–20 mg of *p*Cl-MA as modifier. The content was transferred to mortar and mixed well with plasticizer (0.2 mL of *o*-NPOE, TCP, or DBP) until homogenization of this mixture was achieved, then the resulted paste was used to fill the electrode body, as shown in Table (2).The surface of the resulting CPE was buffed using a filter paper to get a new working surface.

## Results and discussion

### Paste composition and selectivity of the developed carbon sensor

To determine which metal ion, the proposed modifier *p*Cl-MA has a high affinity for it, calibration graphs were constructed for several inorganic cations using the *p*Cl-MA based carbon sensor. As shown in Table 1 the proposed sensor shows a non-Nernstian attitude to all the examined cations except for Al (III) ions.

Several carbon sensors were prepared by mixing different mass ratios of graphite powder, *p*Cl-MA as an electroactive material and TCP as plasticizer, the potential of them was measured as function of Al (III) concentration and constructed in calibration plot. To achieve the repeatability and precision of the measurements, the calibration plot for each composition was repeated 5 times. It is clear from the data listed in Table 2 that the performance of the sensors improved as the amount of *p*Cl-MA increase till the best Nernstian slope of 20.32 ± 1.18 mV decade^− 1^ was obtained by sensor (IV) of composition 2.0% (wt/wt) *p*Cl-MA, 31.24% (wt/wt) TCP and 66.67% (wt/wt) graphite powder, over working concentration range 1.0 × 10^− 6^ to 1.0 × 10^− 1^ mol L^− 1^ of Al(III) with 3.3 × 10^− 7^ mol L^− 1^ as a detection limit. Sensor (IV) was used for subsequent study.

The proportion and nature of the plasticizer is a truly important factor; that must be enhanced to support the optimal activity of the synthesized paste and to eliminate the electrical asymmetry. Several carbon pastes were prepared with different plasticizers, namely, TCP, o-NPOE and DBP to determine the optimum plasticizer integrating in the construction of the proposed electrode. It is clear from the results presented in Table 2, that the sensor plasticized by TCP shows the best trivalent Nernstian slope, reproducibility and good stability; this may be attributed to its high dielectric constant, relatively high molecular weight and the homogenized paste which improve flexibility and durability and diminish the electrical asymmetry of the paste.

Where the proposed sensor achieves Nernstian response, selectivity is a second challenge which might be solved. Theoretically, that the potentiometric selectivity of the sensor is based on the composition of the paste and the complexation specificity of the modifier involved. Depending on the cation size, modifier structure and extractability or complex formation is the determining factor for selectivity^29^. This was confirmed by FT-IR, and UV-spectroscopic analysis.

FT-IR spectrum of the sensor (IV) surface before immersion in aluminum solution was performed and shows the major functional groups of *p*Cl-MA at 1612, 3433, 1214, 1142 and 2924 Cm^− 1^ are assigned to ν (C = O) stretching, ν (O-H) of carboxylic acid, ν (C-O) stretching, ν (C-N-C) asymmetric and ν(C-H) stretching vibration, respectively.

**Fig. 1:**
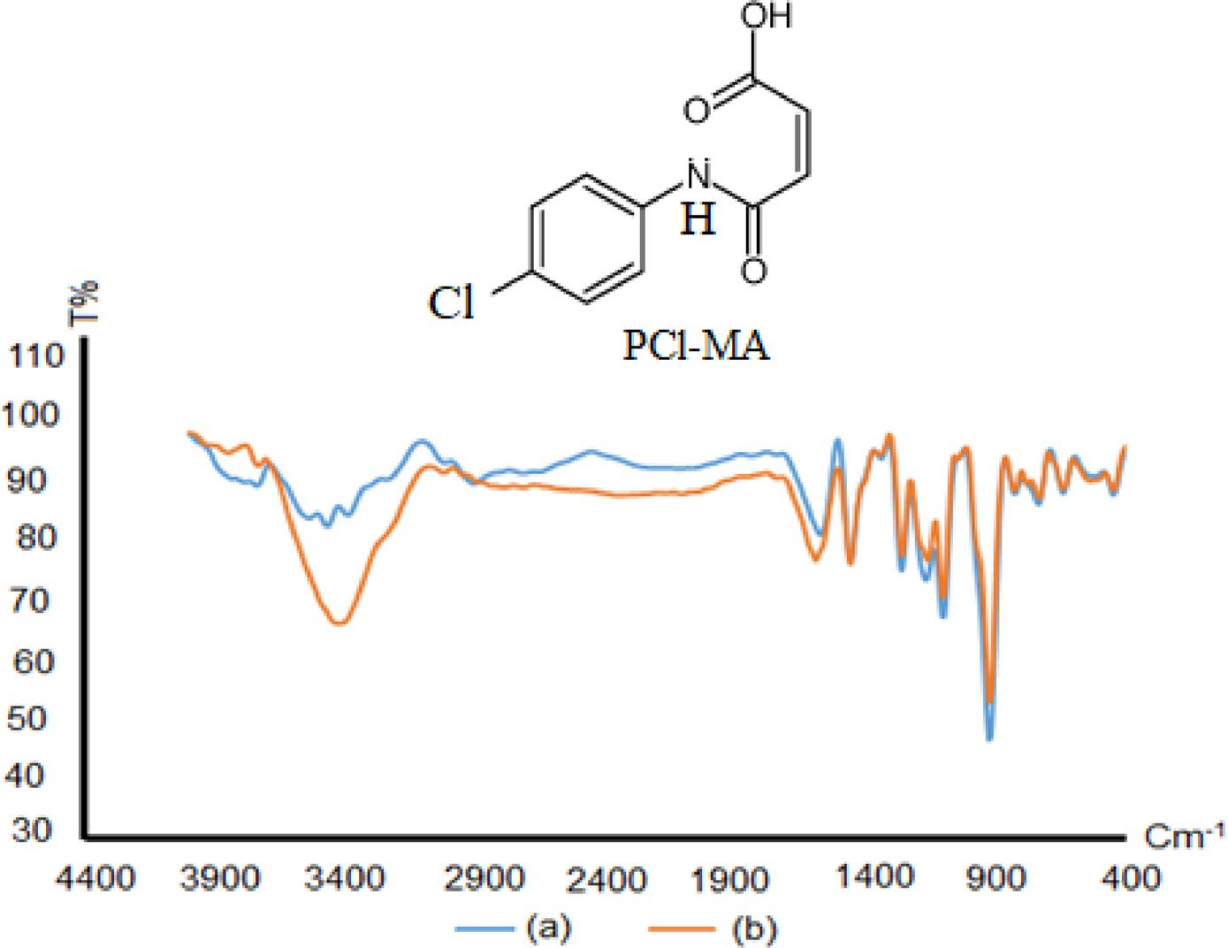
FT-IR of the sensor (IV) (**a**): before and (**b**): after immersion in Al (III) solution.

While after immersing the same sensor surface in Al (III) ion solution, a variation in the FT-IR spectrum was noticed as depicted in Fig. 1. The complex formation takes place through chelation via ν (O-H) of carboxylic and -N- of the amide group (C-N-C) which are shifted to 3480 and 1150 cm^− 1^, respectively^30,31^. Also, the bands due to ν (C-O), ν (C = O) and ν(C-H) were shifted to 1204, 1628 and 2909 cm^− 1^, respectively upon complexation.

**Fig. 2:**
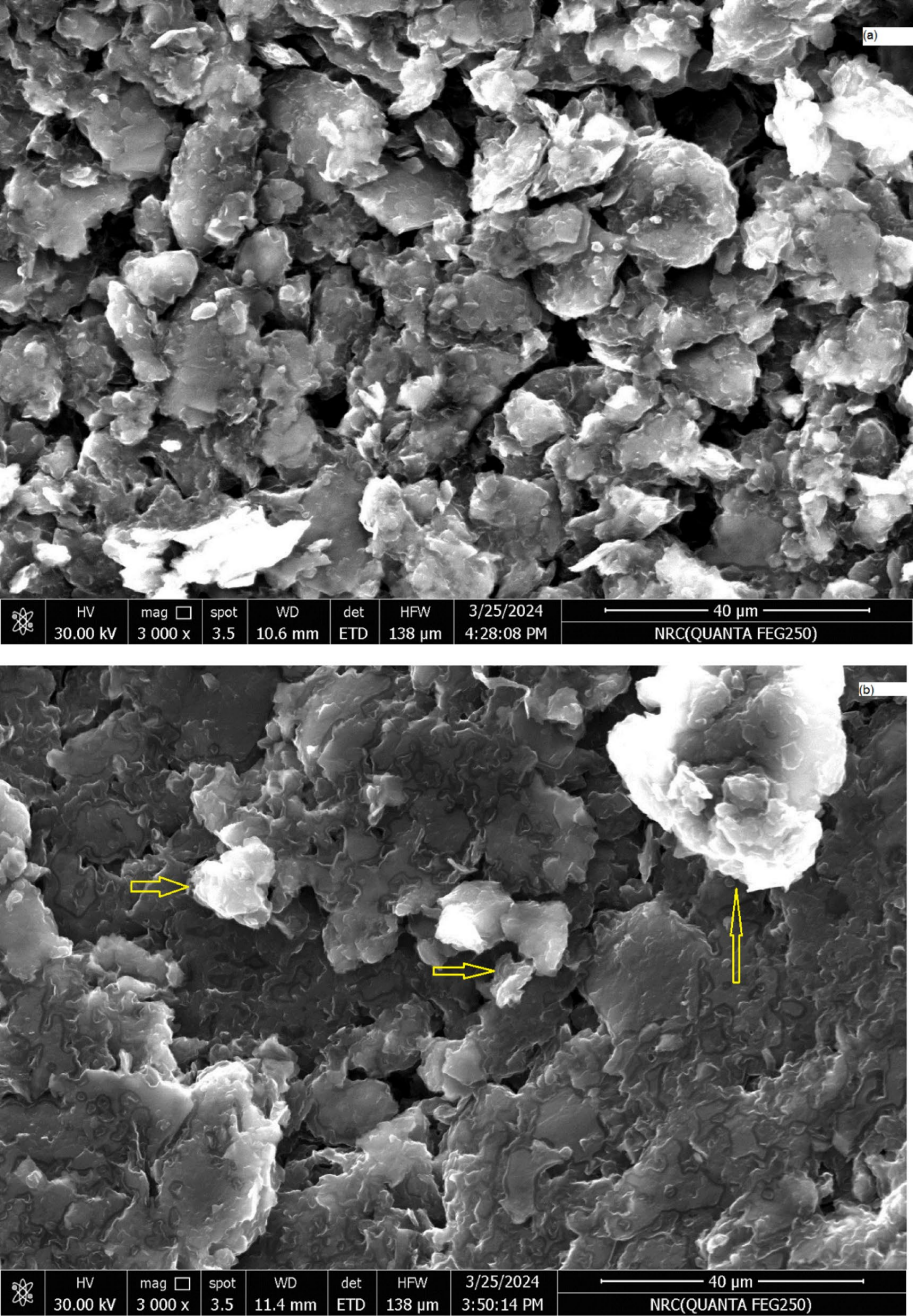
SEM image for the surface of MA-CPE (**a**) before and (**b**) after immersion in Al (III) solution.

**Fig. 3:**
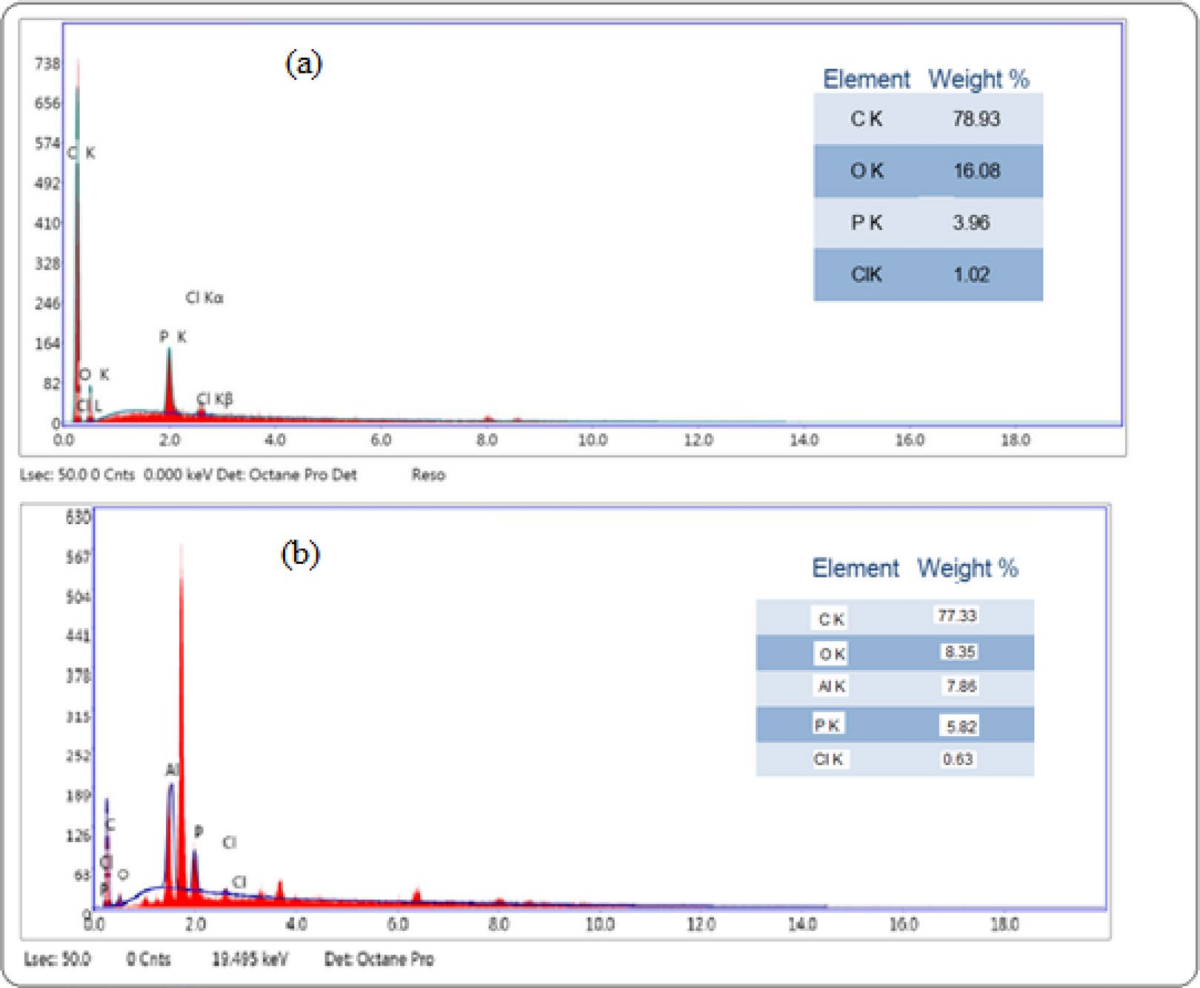
EDX analysis of different elements weight% present in the paste (**a**) before and (**b**) after immersion in aluminum (III) solution.

A complex formed at the surface of carbon sensor by extraction of Al (III) ions from the test solution into the paste phase by the aid of suitable modifier content and plasticizer. This was established by using scanning electron microscope (SEM) and energy dispersive X-ray analysis (EDX) before and after immersion in Al (III) solution. The complex formation was proofed by appearance of brightened spots light the cavities between carbon particles, as shown in Figs. 2 and 3.

The proposed sensor was prepared and its response against the primary Al (III) ion and different cationic species was examined separately (Table 1). Sensor’s selectivity can be powerfully expressed in terms of selectivity coefficients, and they were determined by separate solution method (SSM)^32,33^. If the value of selectivity coefficient equal to 1.0 indicates equal response to both primary ion and interfering ions, while if the value of selectivity coefficient is smaller than 1.0 it shows that the sensor is selective to the primary ion over the interfering ions. It is obvious from the data presented in Table 1, that the selectivity coefficients values are very small in most examined cations, which proved high selectivity of the proposed sensor toward Al (III) ions over other cations. This can be attributed to the selectivity, interaction between *p*Cl-MA ionophore and Al (III) ion; also, due to the fast exchange^34,35^.

**Table 1:** The potentiometric selectivity coefficients of sensor (IV) using SSM, slope values, working concentration range and correlation coefficient of calibration curves of some interfering ions.

Interfering Species	Slope mV decade^− 1^	correlation coefficient, *r*^2^	Linear concentration range, mol L^− 1^	Log K^pot^_Al_^3+^_, B_
Al(III)	**20.32**	**0.9972**	**1.0 × 10**^**−6**^ : **1.0 × 10**^**−1**^	**-**
Cu(II)	33.3	0.9953	1.0 × 10^−5^ : 1.0 × 10^−2^	−6.394
Mn(II)	22.7	0.9740	1.0 × 10^−6^ : 1.0 × 10^−1^	−5.242
Sr(II)	16.1	0.9916	1.0 × 10^−6^ : 1.0 × 10^−2^	−9.032
Ca(II)	11.2	0.9299	1.0 × 10^−7^ : 1.0 × 10^−2^	0.663
Pb(II)	18.0	0.9959	1.0 × 10^−6^ : 1.0 × 10^−4^	1.549
Co(II)	8.5	0.7773	1.0 × 10^− 6^ : 1.0 × 10^−3^	−6.128
Ni(II)	7.1	0.9669	1.0 × 10^−4^ : 1.0 × 10^−1^	−6.226
Fe(III)	7.0	0.9423	1.0 × 10^−7^ : 1.0 × 10^−5^	6.077
Mo (VI)	2.2	0.7749	1.0 × 10^−7^ : 1.0 × 10^−1^	−7.750

**Table 2:** Effect of *p*Cl-MA content and plasticizer type on the performance of the carbon sensors for potentiometric calibration of al (III) ion.

Sensor no.	Paste composition % wt/wt				Linear range (mol L^− 1^)	Slope, mv decade^− 1^	Correlation coefficient (*r*^2^)
	Graphite powder	Ionophore (PCl-MA)	plasticizer	Plasticizer type			
I	68.11	0.00	31.88	TCP	1.0 × 10^− 5^ − 1.0 × 10^− 1^	8.33 ± 0.321	0.9991
II	67.66	0.677	31.66	TCP	1.0 × 10^− 6^ − 1.0 × 10^− 2^	19.18 ± 2.44	0.9903
III	67.2	1.344	31.45	TCP	1.0 × 10^− 6^ – 5.0 × 10^− 2^	19.56 ± 1.50	0.9965
**IV**	**66.76**	**2.00**	**31.24**	**TCP**	**1.0 × 10**^**− 6**^ **− 1.0 × 10**^**− 1**^	**20.32 ± 1.18**	**0.9972**
V	66.31	2.65	31.03	TCP	1.0 × 10^− 6^ − 1.0 × 10^− 1^	22.56 ± 1.44	0.9960
VI	68.72	2.06	29.22	o-NPOE	1.0 × 10^− 6^ − 1.0 × 10^− 1^	19.45 ± 0.826	0.9985
VII	70.22	2.11	27.67	DBP	1.0 × 10^− 4^ – 1.0 × 10^− 1^	27.51 ± 1.32	0.9988

### pH effect

**Fig. 4:**
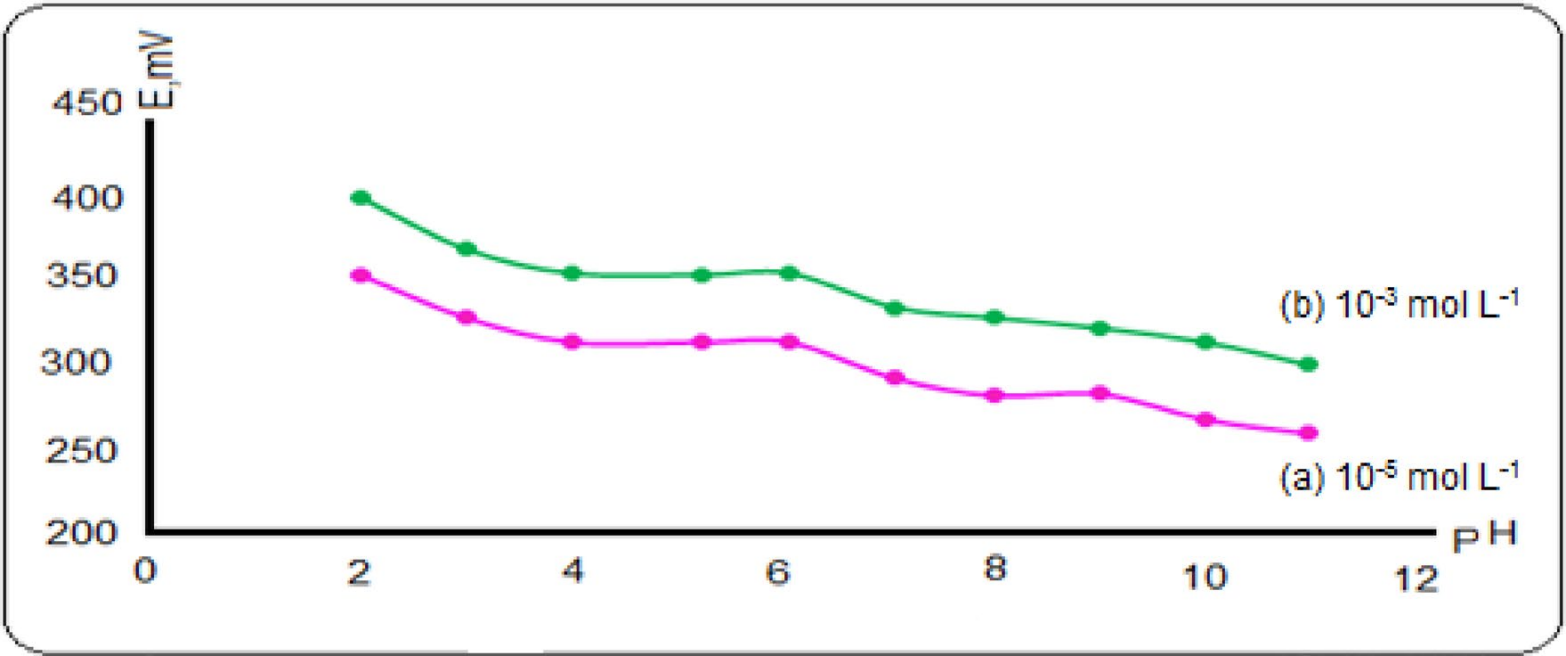
Effect of pH on potential response of the sensor (IV) (**a**): 1 × 10^− 5^ mol L^− 1^ Al (III) and (**b**): 1 × 10^− 3^ mol L^− 1^ Al (III).

The pH effect on the proposed Al (III) sensor was examined in the pH range of 2–11 on 1.0 × 10^− 5^ and 1.0 × 10^− 3^ mol L^− 1^ of Al (III) solutions. The potential measured by the proposed sensor (IV) was plotted against the pH of Al (III) ions solution as presented in Fig. 4. From the Figure it is found that the potential constant over pH is from 4 to 6. The potential readings increase at pH less than 4 due to response of the sensor to the H^+^ ion in addition to Al (III) ions in the solution. While the observed decline in the potential at pH greater than 6, may be attributed to hydrolysis of Al (III) ions and formation of some insoluble or soluble hydroxide^36,37^.

### Effect of temperature

The effect of temperature on the performance of the developed sensor was studied by constructing the potential calibration graphs in temperature range from 10 to 60 °C, to select the optimum temperature for the aluminum determination.

The isothermal coefficient (dE°/dt) cell was calculated by plotting the standard electrode potential against (t-25) using Antropov’s equation^38^. It is found to be 4.291 × 10^− 3^ V °C^− 1^. According to the isothermal coefficient value the sensor exhibited excellent thermal stability up to 60 °C without deviating from the trivalent Nernstian slope.

### Response time

**Fig. 5:**
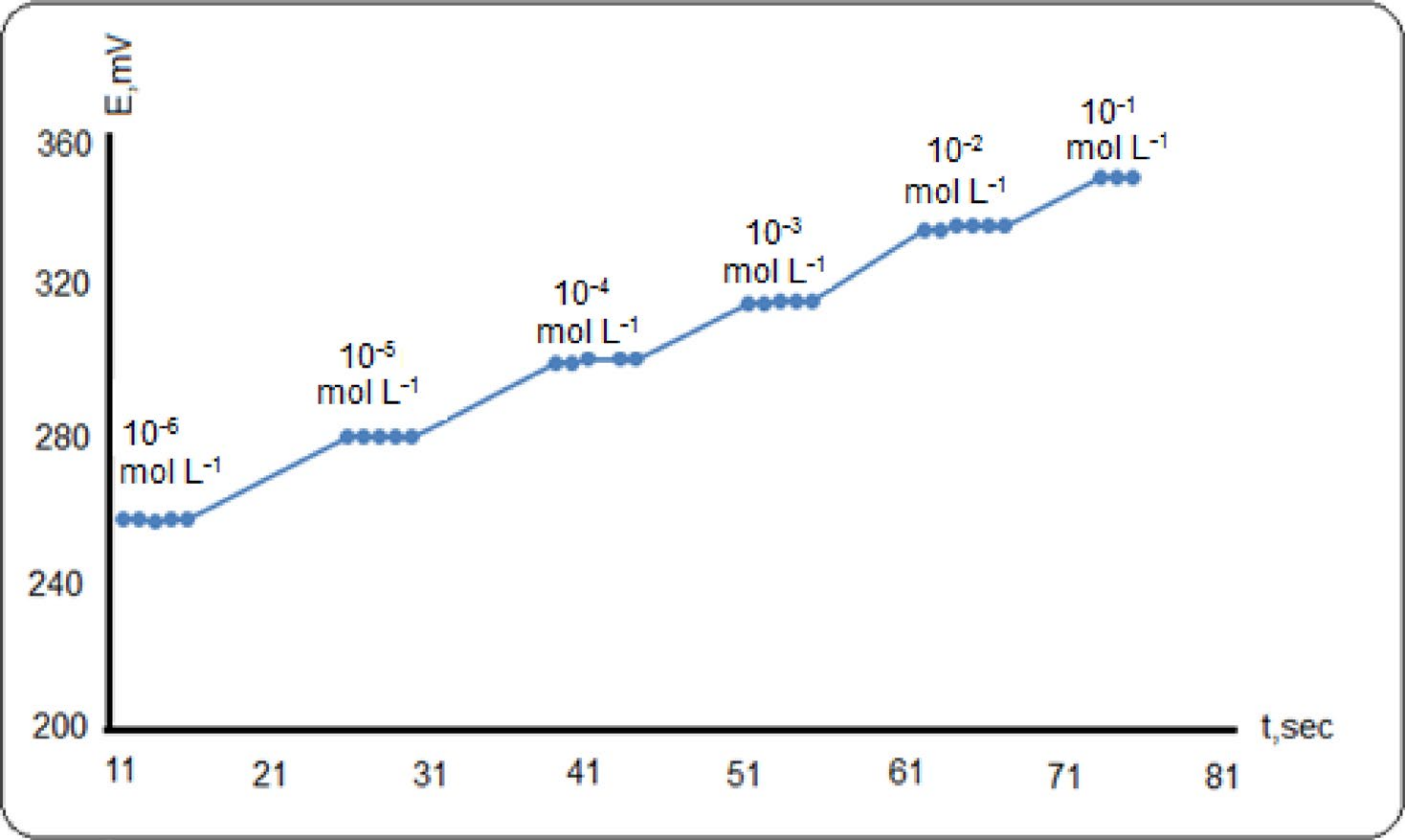
Dynamic response of sensor (IV).

The response time of the sensor can be expressed as the average time of the sensor’s needed to reach steady state potential reading^39^. Dynamic response of ion selective electrode is generated by selective complexation of the *p*Cl-MA as neutral carrier molecule dispersed in a paste matrix to the primary ion, Al (III). The response time of the proposed carbon sensor was measured through the immersion of the carbon sensor in conjugation with the reference electrode in aluminum solution which has been successively changed from lower concentration 1.0 × 10^− 6^ to higher concentration 1.0 × 10^− 1^ mol L^− 1^. The potential readings were plotted against time as graphically represented in Fig. 5. It was found that the average response time of the sensor (IV) is 8 s which reflect the fast exchange kinetics of complexation–decomplexation of aluminum ions with the *p*Cl-MA at the paste- test solution interface.

### Analytical applications

The proposed carbon sensor was applied for the micro- determination of Al (III) ions concentrations in a variety of real samples including pharmaceutical formulation, soft drink and biological fluids to prove the applicability of the selected method.

The proposed electrode is shown to be sufficiently useful in the direct potentiometric determination of Al (III) in Maalox using calibration method.

The Al (III)-carbon sensor based on *p*Cl-MA was used to determine the amount of Al (III) in the soft drink which is packed in cane and plastic bottle. The results indicated the elevation of aluminum level in the soft drink which was packed in cane because of the aluminum swept from cane to it.

Moreover, the proposed sensor was successfully applied to determine the concentration of Al (III) in different serum samples of kidney failure and Alzheimer disease patients, which are expected to have high levels of aluminum.

The obtained results indicated great agreement between the data obtained by the proposed Al-carbon sensor based on *p*Cl-MA with those obtained by ICP as presented in Table 3.

**Table 3:** Determination of al (III) ion in different real samples; pharmaceutical formulation, beverage cane and human serum of kidney failure and alzheimer disease patients using sensor (IV).

Real sample	Found, ppm		t- test
	Carbon sensor	ICP	
Maalox	274.5	270	0.636
Cane	0.1484	0.1518	0.835
Plastic bottle	0.027>	0.003	-
Patient 1	37.34	39.96	2.50
Patient 2	33.34	34.02	0.65
Patient 3	39.82	39.96	0.164

Tabulated t- values at 95% confidence level is 2.571 (*n* = 5).

### Comparison study

The proposed sensor presented an acceptable and good performance when compared with the previously published Al (III) - ISE concerning linear ranges Nernstian slope, limit of detection (LOD), response time and pH, as shown in Table 4.

**Table 4:** Comparison between the proposed sensor and previously published Al(III)- selective electrodes.

Electrode type	Modifier	Slope, mVdecade^− 1^	Linear range mol L^− 1^	pH range	Response time (sec)	LOD mol L^− 1^	Ref
PVC	12-crown-4 (12C4)	19.0 ± 0.4	1 × 10^− 6^−1 × 10^− 2^	4.0–8.0	15.0	5.5 × 10^− 7^	^18^
all-solid-state	1,10[(methylazanediyl)bis(ethane-2,1-diyl)]bis[3-(naphthalen-1-yl)thiourea]	17.7 ± 0.13	1 × 10^− 6^−1 × 10^− 2^	2.0–8.0	> 50.0	2.45 × 10^− 7^	^40^
PVC	Hydroxy -thioxanthone derivatives	21.50	2 × 10^− 6^- 2 × 10^− 2^	3.4–5.0	5.0	1 × 10^− 6^	^41^
PVC	Bis (5-sulphonate salicylaldehyde) 2,3- diaminobenzene	15.6	1 × 10^− 5^−1.5 × 10^− 1^	3.5–4.5	-	5.1 × 10^− 6^	^17^
PVC	Zinc(II) complex of Thiophenealdehydethiosemicarbazone [Zn(LL_2_)_2_]Cl_2_	19.8 ± 0.1	1.0 × 10^− 7^−1 × 10^− 1^	2.4–9.5	> 10.0	6.7 × 10^− 9^	^19^
PVC	5,10,15,20-tetrakis(p-chlorophenyl)porphyrin	25.0 ± 2.7	1.09 × 10^− 5^−1.09 × 10^− 1^	5.0–10.0	> 5.0	2.81 9 10^− 6^	^42^
CPE	Flubendazole drug	20.11 ± 0.47	1.0 × 10^− 7^ −1.0 × 10^− 1^	3.0–5.0	4.0	1.0 × 10^− 7^	^5^
CPE	1,8-dihydroxyanthraquinone (DHAQ)	20.7 ± 0.5	1 × 10^− 6^– 1 × 10^− 1^	2.0–5.0	20.0	5.0 × 10 − 7	^43^
CPE	Carboxymethyl chitosan-graft-poly(1-cyanoethanoyl-4-acryloyl-thiosemcarbazide) copolymers	19.9 ± 0.36	1 × 10^–6^ − 1 × 10^–2^	3.0–8.0	4.5	1 × 10^–6^	^44^
CPE	N, N’-bis(salicylidene)−1,3-Propanediamine	20.2 ± 0.1	1.0 × 10^–6^- 1.0 × 10^–2^	2.0–4.0	5	1 × 10^–6^	^45^
CPE	bis(tert-butylaminomethyl)pyridine pincer	20.05 ± 0.27	1.0 × 10^− 7^ −1.0 × 10^− 1^	2.5–5.5	5	1.0 × 10^− 7^	^46^
SPE	Quercetin	21.9 ± 0.4	2.3 × 10^− 8^ −1.07 × 10^− 3^	3.0–5.0	3	2.3 × 10^− 8^	^47^
ISE	boron nitride (BN) nanoparticles	22.05	1.0 × 10⁻⁷−1.0 × 10^− 1^		10	7.5 × 10 ^−8^	^48^
CPE	*p*Cl-MA	20.32 ± 1.18	1 × 10^− 6^ −1 × 10^− 1^	4.0–7.0	8.0	3.3 × 10^− 7^	This work

## Conclusion

Al (III)-carbon sensor based on p-chlorophenyl maleanilic acid (*p*Cl-MA) was developed. The proposed sensor exhibits precise, fast response of 8 s., reproducible and selective potentiometric investigation of aluminum ions. It shows trivalent Nernstian slope (20.32 ± 1.18) over a linear range 1.0 × 10^− 6^ −1.0 × 10^− 1^ mol L^− 1^ and high selectivity toward aluminum ions over the other cationic species. Moreover, the developed sensor successfully applied to detect the trace level of aluminum in real samples such as pharmaceutical, canned beverages and biological fluids of kidney failure and Alzheimer disease patients, without the need to complicated instrumentation or extra separation techniques like coprecipitation and ion extraction.

## Supplementary Information

Below is the link to the electronic supplementary material.

Supplementary Material 1

## Data Availability

The data supporting this article has been included as part of the manuscript.
