# Supplementary material for: Quantitative determination of al (III) traces in soft drink, pharmaceutical products and biological fluids of kidney failure and alzheimer disease patients using carbon sensor
Source: Sci Rep. 2026 Jan 6;16:626. doi: 10.1038/s41598-025-32308-z (PMC12774894; doi:10.1038/s41598-025-32308-z)
Supplement: Supplementary file 1 — Supplementary Material 1 [file 41598_2025_32308_MOESM1_ESM.docx]

**Supplementary material**

**Quantitative determination of Al (III)** **traces in soft drink, pharmaceutical products and biological fluids of kidney failure and Alzheimer disease patients** **using carbon sensor**

M. H. Abdel Basset^1^, M. A. Zayed^1^, Eman Yossri Frag^1^*.

^1^Chemistry Department, Faculty of Science, Cairo University, Gamaa Str., 12613, Giza, Egypt.

*Corresponding author: Eman Yossri Frag

E-mail: (emanyossri@sci.cu.edu.eg)

**Fig. S1** Calibration curves of Al (III) ion solution using sensor (IV).
